# Supplementary material for: Chord: an ensemble machine learning algorithm to identify doublets in single-cell RNA sequencing data
Source: Commun Biol. 2022 May 30;5:510. doi: 10.1038/s42003-022-03476-9 (PMC9151659; doi:10.1038/s42003-022-03476-9)
Supplement: Supplementary file 2 — Supplementary Information [file 42003_2022_3476_MOESM2_ESM.pdf]

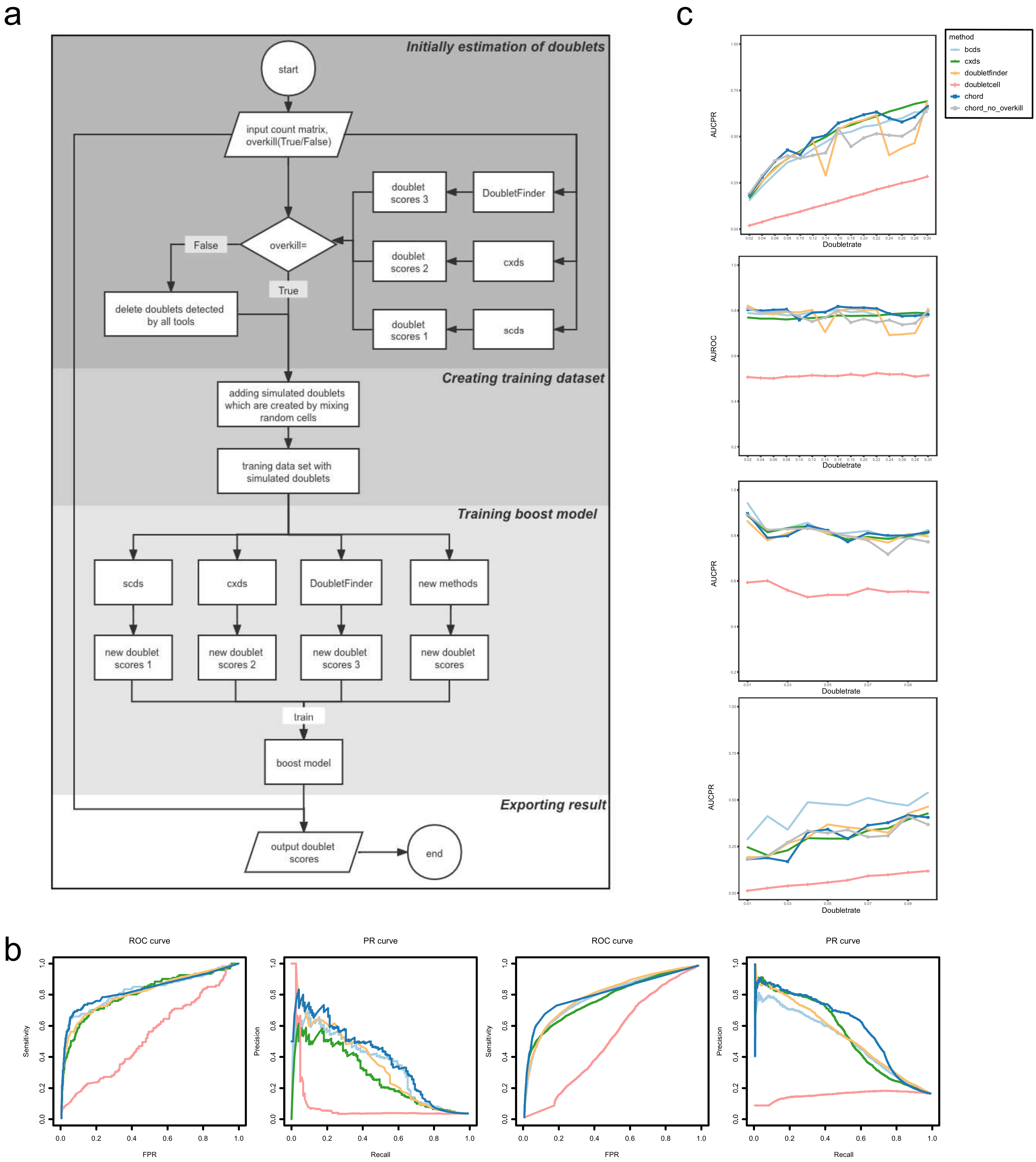

Supplementary Figure 1. The flow chart of Chord

(a) The workflow of the Chord software is divided into four parts. The flow chart shows the data flow of the software.
(b) The receiver operating characteristic curves (ROC) and PR curve of Chord and the other four methods were draw for the test DM-A (the first row) and HTO8 (the second row).
(c) We generated the HTO8 sub-datasets with doublet rate from 0.02 to 0.30 (the first two from the top) by randomly sampling the dataset, and the sub-datasets with doublet rate from 0.01 to 0.10 of the data set DM-A (the third and fourth from the top). The values of AUC and PAUC on these datasets are calculated respectively for Chord, Chord (overkill=F) and the other four methods.

a

DM-A

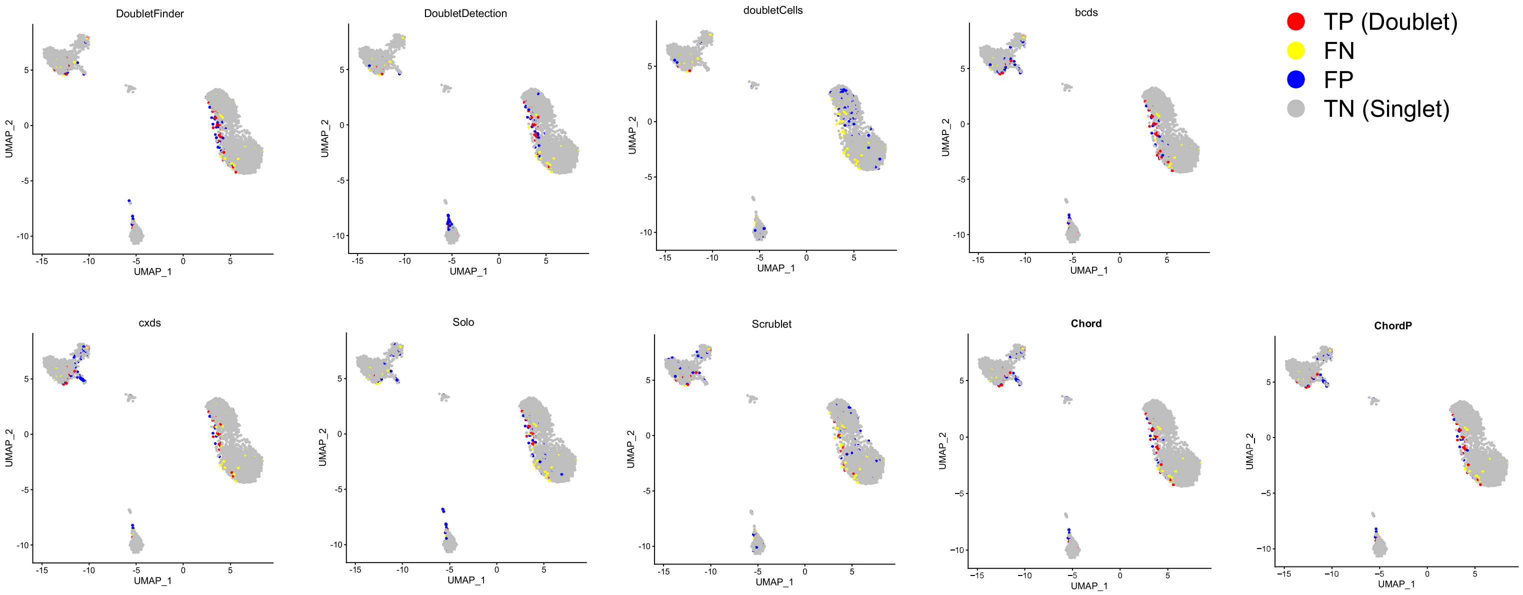

b

HTO8

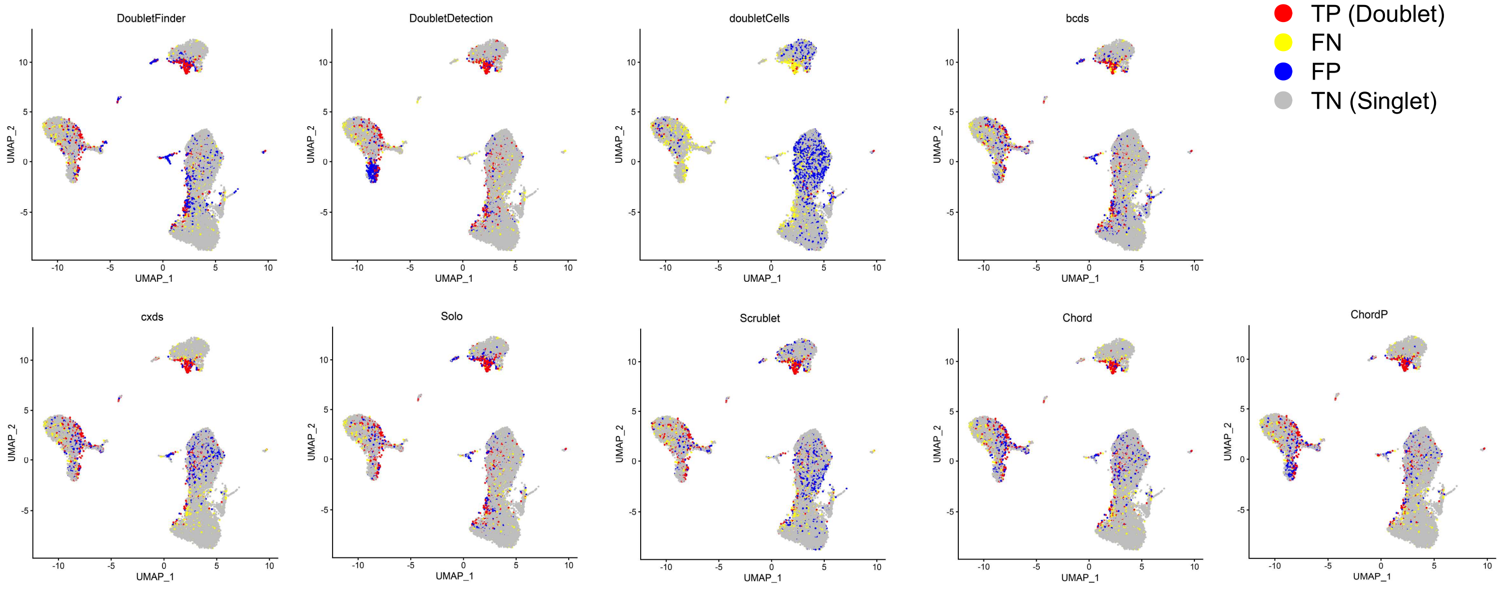

c

DM-A

	TP	FN	FP	TN	TPR	TNR	Precision	Accuracy
bcds	52	68	68	3110	0.4333	0.9786	0.4333	0.9588
cxds	46	74	74	3104	0.3833	0.9767	0.3833	0.9551
Chord	56	64	64	3114	0.4667	0.9799	0.4667	0.9612
ChordP	55	65	65	3113	0.4583	0.9795	0.4583	0.9606
doubletCells	11	109	109	3069	0.0917	0.9657	0.0917	0.9339
DoubletFinder	51	69	69	3109	0.425	0.9783	0.425	0.9582
DoubletDetection	45	75	75	3103	0.375	0.9764	0.375	0.9545
Scrublet	43	77	77	3101	0.3583	0.9758	0.3583	0.9533
Solo	32	88	88	3090	0.2667	0.9723	0.2667	0.9466

HTO8

	TP	FN	FP	TN	TPR	TNR	Precision	Accuracy
bcds	1349	1249	1249	12723	0.5192	0.9106	0.5192	0.8492
cxds	1369	1229	1229	12743	0.5269	0.912	0.5269	0.8517
Chord	1596	1002	1002	12970	0.6143	0.9283	0.6143	0.8791
ChordP	1642	956	956	13016	0.632	0.9316	0.632	0.8846
doubletCells	264	2334	2334	11638	0.1016	0.833	0.1016	0.7183
DoubletFinder	1349	1249	1249	12723	0.5192	0.9106	0.5192	0.8492
DoubletDetection	1594	1004	1004	12968	0.6135	0.9281	0.6135	0.8788
Scrublet	1291	1307	1307	12665	0.4969	0.9065	0.4969	0.8422
Solo	1712	886	886	13086	0.659	0.9366	0.659	0.8931

Supplementary Figure 2. UMAP visualization of the DM-A and HTO8 datasets in terms of the doublet, singlet, FP and FN results.

(a, b) For the real dataset DM-A (Figure.S2a) and dataset HTO8 (Figure.S2b), according to the scoring results of each method, we deleted the top scoring cells according to the real doublet rate, and verified with the real label to visually show the true positive (TP) doublet, true positive (TN) singlet, false positive (FP) and false negative (FN) results.
(c) TP,FN,FP,TN,True Positive Rate(TPR),True Negative Rate(TNR),Precision,Accuracy in dM-A (left) and HTO8(right) data sets

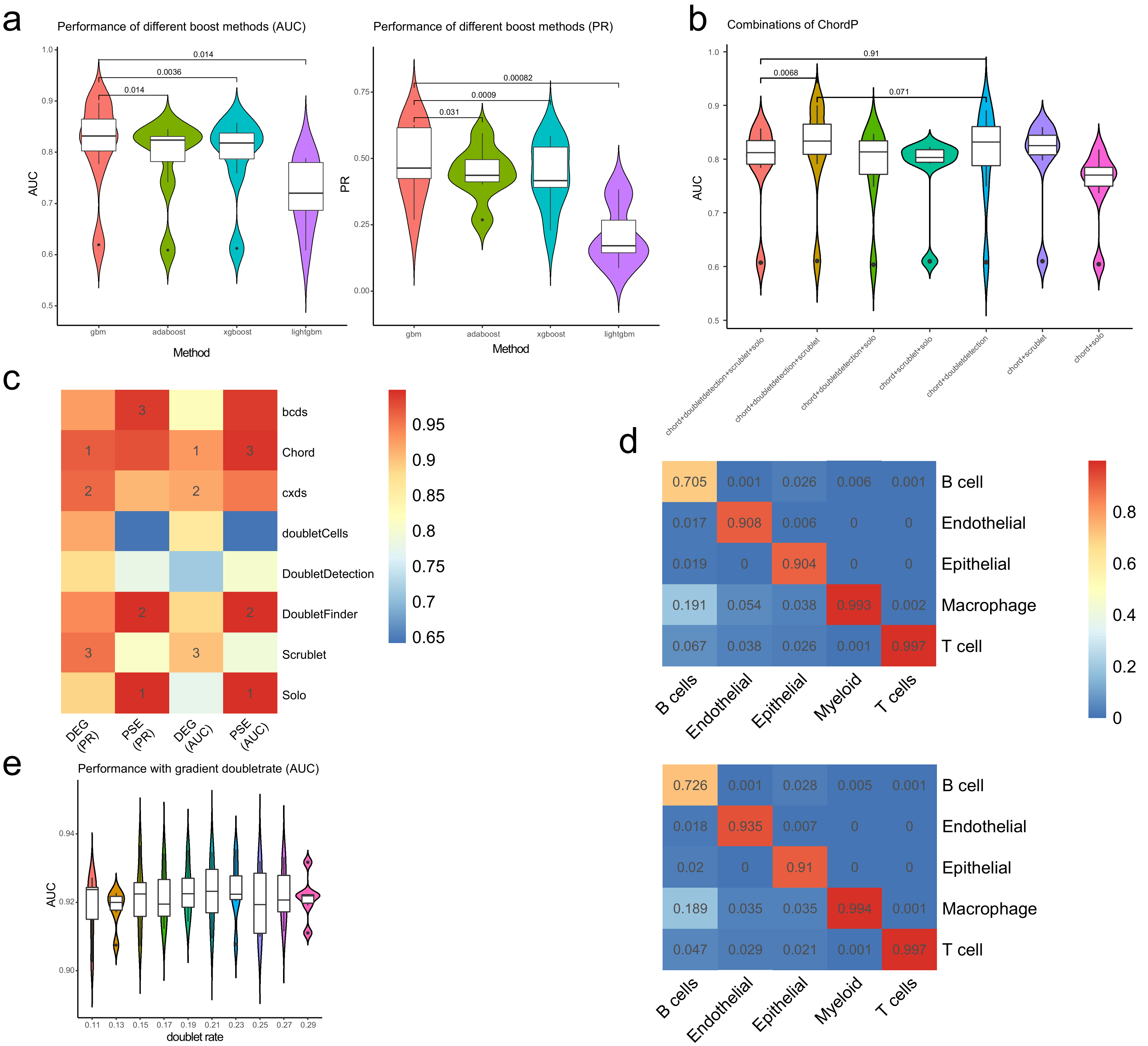

Supplementary Figure 3. Parameter selection for Chord, and combination evaluation for ChordP.
(a) Chord's performance when using different boost algorithms. The evaluation data sets consist of HTO8, HTO12, DM-A, DM-B, DM-C, DM-2.1, DM-2.2 (paired t-test).
(b) The mean AUC of all combinations of ChordP across benchmarking datasets. The evaluation datasets consist of HTO8, HTO12, DM-A, DM-B, DM-C, DM-2.1, DM-2.2 (paired t-test).
(c) The heatmap of the PR and AUC values of the eight methods on the PSE and DEG datasets. The number in the figure indicates the rank of the method in the dataset (only the top three methods are marked).
(d) Automated cell type annotation of this data set was performed using SciBet. The training model provided by SciBet, which was trained from 42 human single-cell datasets containing 30 major human immune cell types, was used to automatically annotate the datasets before and after doublet removal. The matrix heatmaps are plotted with the SciBet scores.
(e) Chord's performance with different parameter doubletrate from 0.11 to 0.29 on six data sets. These 6 datasets were randomly sampled and generated by the DEG dataset, and the true doublet rate was 0.2.

Supplementary Table 1. Overview of the computational methods for doublet detection and their capabilities					
Method	Platform	Category	Model Description	Version	Reference
DoubletFinder[1]	R	simulate artificial doublets	use simulated doublet generates simulated doublets and add them to original cells. on the basis of the fraction of simulated doublets in the neighborhood of each cell, calculating scores by pANN.	2.0.3	McGinnis et al. (2019a)
scrublet[2,3]	Python	simulate artificial doublets	Generate simulated doublet data, cluster together with the original cells.in the principal component (PC) space, the proximity of cells to the doublet was evaluated by KNN algorithm.	0.2.1	Wolock et al. (2019)
doubletCells[4] (scran)	R	simulate artificial doublets	It simulates doublets by adding two randomly selected cells, and calculates the proportion of simulated doublets of every cell to define the cells which are closed to many simulated doublets as doublets.	1.16.0	Lun et al., 2016
bcdx[5]	R	simulate artificial doublets	It generates simulated doublets by adding two randomly selected cells gene expression. Then mixing these simulated doublets with the original cells, and trains a gradient boosting classifier to classify the mixed cells into simulated doublets and original cells. The scores of each cell are defined as the frequency of being classified to simulated doublets.	1.4.0	A.S.Bais and D.Kostka.2019
cxdx[5]	R	caculate marker gene pair	It calculates a p value for each pair of genes under the null hypothesis that the number of cells where exactly one of the two genes is expressed follows a binomial distribution, then it defines co-expressed gene pairs which are mostly expressed in doublets, and classify the doublets and singlets by the expression of co-expressed gene pairs.	1.4.0	A.S.Bais and D.Kostka.2020
solo[6]	Python	simulate artificial doublets	Gene distribution was estimated according to randomly sampled cells, and then gene expression profile was extracted randomly to synthesize doublets. Combine the doublets with the original data and train the neural network to recognize.		Nicholas J. Bernstein et 2020
DoubletDetection[6]	Python	simulate artificial doublets	A small number of simulated twin cells were generated by randomly sampling cells, and the possible doublets were calculated based on the distance algorithm. Then it executed a iterative calculation.	2.5.2	Gayoso and Shor, 2018

Supplementary Table 2. Overview of the real scRNA-seq datasets with experimentally annotated doublets used in the study.

Dataset	Experimental method	Species	Tissue	Number of cells	Number of doublets	Median UMI count	Median gene count	doublet rate
Kang et al. Control PBMCs (DM-2.1) [7]	Demuxlet	Human	PBMCS	14619	1512	1256	520	10.343
Kang et al. Stimulated PBMCs (DM-2.2) [7]	Demuxlet	Human	PBMCS	14446	1552	1345	546	10.743
Kang et al. A PBMCs (DM-A) [7]	Demuxlet	Human	PBMCS	3298	120	973	384	3.639
Kang et al. B PBMCs (DM-B) [1]	Demuxlet	Human	PBMCS	3790	130	862	361	3.430
Kang et al. C PBMCs (DM-C) [7]	Demuxlet	Human	PBMCS	5270	316	829	352	5.996
Stoeckius et al. Cell lines (HTO12) [8]	Cell Hashing (ACOs)	Human	PBMCS	8191	889	4636	2086	10.853
Stoeckius et al. Cell lines (HTO8) [8]	Cell Hashing (ACOs)	Human	PBMCS	15583	2545	550	321	16.332

Supplementary Table 3. AUC relative to DM-A dataset in Supplementary Figure 1b						
rate	bcds	cxds	doubletfinder	doubletcell	chord	chord_no_overkill
0.01	0.943	0.888	0.863	0.593	0.897	0.893
0.02	0.816	0.815	0.779	0.601	0.789	0.824
0.03	0.833	0.833	0.809	0.559	0.798	0.829
0.04	0.856	0.841	0.836	0.529	0.844	0.831
0.05	0.806	0.808	0.810	0.539	0.823	0.819
0.06	0.812	0.782	0.798	0.539	0.772	0.796
0.07	0.821	0.794	0.788	0.567	0.810	0.779
0.08	0.793	0.785	0.769	0.552	0.800	0.717
0.09	0.788	0.797	0.806	0.555	0.801	0.789
0.1	0.824	0.810	0.797	0.550	0.816	0.772

Supplementary Table 4. AUC relative to HTO8 dataset in Supplementary Figure 1b						
rate	bcds	cxds	doubletfinder	doubletcell	Chord	chord_no_overkill
0.02	0.789	0.769	0.821	0.507	0.803	0.809
0.04	0.784	0.764	0.790	0.503	0.799	0.791
0.06	0.786	0.765	0.783	0.501	0.802	0.793
0.08	0.779	0.761	0.794	0.509	0.805	0.797
0.12	0.793	0.767	0.803	0.515	0.791	0.751
0.14	0.794	0.770	0.705	0.512	0.793	0.769
0.16	0.803	0.778	0.799	0.512	0.818	0.800
0.18	0.793	0.776	0.807	0.519	0.812	0.747
0.1	0.781	0.765	0.792	0.510	0.758	0.772
0.22	0.779	0.777	0.803	0.525	0.808	0.749
0.24	0.781	0.784	0.692	0.519	0.786	0.758
0.26	0.779	0.787	0.695	0.520	0.775	0.737
0.28	0.779	0.790	0.701	0.509	0.777	0.744
0.2	0.796	0.777	0.801	0.513	0.812	0.763
0.3	0.772	0.788	0.807	0.514	0.783	0.799

Supplementary Table 5. PR relative to DM-A dataset in Supplementary Figure 1b						
rate	bcds	cxds	doubletfinder	doubletcell	chord	chord_no_overkill
0.01	0.289	0.247	0.193	0.013	0.182	0.182
0.02	0.413	0.204	0.192	0.027	0.189	0.200
0.03	0.341	0.230	0.267	0.039	0.169	0.272
0.04	0.487	0.295	0.299	0.046	0.327	0.333
0.05	0.478	0.292	0.368	0.057	0.342	0.322
0.06	0.470	0.293	0.353	0.069	0.292	0.340
0.07	0.511	0.335	0.342	0.093	0.364	0.301
0.08	0.485	0.348	0.324	0.098	0.378	0.307
0.09	0.470	0.395	0.427	0.111	0.419	0.411
0.1	0.539	0.428	0.464	0.118	0.406	0.368

Supplementary Table 6. PR relative to HTO8 dataset in Supplementary Figure 1b						
rate	bcds	cxds	doubletfinder	doubletcell	chord	chord_no_overkill
0.02	0.156	0.168	0.185	0.020	0.183	0.191
0.04	0.231	0.258	0.259	0.038	0.280	0.289
0.06	0.297	0.330	0.324	0.060	0.365	0.369
0.08	0.360	0.382	0.383	0.076	0.427	0.397
0.12	0.431	0.464	0.477	0.115	0.491	0.399
0.14	0.470	0.497	0.290	0.133	0.507	0.412
0.16	0.514	0.542	0.538	0.152	0.573	0.543
0.18	0.526	0.562	0.574	0.173	0.594	0.446
0.1	0.386	0.422	0.415	0.095	0.403	0.382
0.22	0.563	0.609	0.617	0.214	0.633	0.516
0.24	0.587	0.635	0.400	0.230	0.600	0.507
0.26	0.598	0.655	0.437	0.249	0.579	0.502
0.28	0.632	0.677	0.465	0.263	0.606	0.544
0.2	0.554	0.588	0.590	0.190	0.618	0.493
0.3	0.633	0.691	0.691	0.284	0.663	0.651

Supplementary Table 7. AUC relative to combinations of ChordP in Supplementary Figure 3b

	chord+doubletdetection+scrublet+solo	chord+doubletdetection+scrublet	chord+doubletdetection+solo	chord+scrublet+solo	chord+doubletdetection	chord+scrublet	chord+solo
MD-A	0.820	0.833	0.824	0.822	0.832	0.831	0.818
DM-B	0.784	0.791	0.748	0.791	0.754	0.798	0.742
DM-C	0.812	0.827	0.813	0.796	0.826	0.818	0.778
DM-2.1	0.849	0.896	0.841	0.816	0.887	0.858	0.763
DM-2.2	0.857	0.900	0.847	0.820	0.890	0.860	0.770
HTO12	0.607	0.610	0.603	0.610	0.608	0.610	0.604
HTO8	0.797	0.835	0.795	0.803	0.834	0.825	0.791
Mean AUC	0.789	0.813	0.782	0.780	0.805	0.800	0.752

Supplementary Table 8. AUC relative to parameter doubletrate in Supplementary Figure 3e											
	0.11	0.13	0.15	0.17	0.19	0.21	0.23	0.25	0.27	0.29	correlation	p-value
data1	0.912	0.921	0.920	0.922	0.914	0.928	0.930	0.910	0.921	0.922	0.224	0.533
data2	0.938	0.942	0.933	0.934	0.928	0.935	0.935	0.930	0.935	0.934	-0.419	0.228
data3	0.899	0.904	0.895	0.901	0.908	0.908	0.900	0.911	0.910	0.923	0.777	0.008
data4	0.919	0.915	0.906	0.916	0.914	0.921	0.926	0.911	0.907	0.860	-0.520	0.123
data5	0.935	0.942	0.936	0.937	0.933	0.939	0.931	0.936	0.929	0.927	-0.703	0.023
data6	0.922	0.933	0.933	0.931	0.929	0.928	0.927	0.931	0.926	0.928	-0.094	0.797
mean AUC	0.921	0.926	0.921	0.924	0.921	0.927	0.925	0.921	0.921	0.916	-0.380	0.279

Supplementary Table 9. Data source		
Resource	Source	Location
Control PBMCs (DM-2.1)	Kang *et al.*,2018	downloaded from the GEO with the accession GSE96583
Stimulated PBMCs (DM-2.2)	Kang *et al.*,2018	downloaded from the GEO with the accession GSE96583
A PBMCs (DM-A)	Kang *et al.*,2018	downloaded from the GEO with the accession GSE96583
A PBMCs (DM-B)	Kang *et al.*,2018	downloaded from the GEO with the accession GSE96583
A PBMCs (DM-C)	Kang *et al.*,2018	downloaded from the GEO with the accession GSE96583
A PBMCs (HTO12)	Stoeckius *et al.*,2018	https://www.dropbox.com/sh/ntc33ium7cg1za1/AAD_8XIDmu4F7lJ-5sp-rGFYa?dl=0
A PBMCs (HTO8)	Stoeckius *et al.*,2018	https://www.dropbox.com/sh/ntc33ium7cg1za1/AAD_8XIDmu4F7lJ-5sp-rGFYa?dl=0
DEG test dataset	Nan Miles Xi *et al.*,2019	https://zenodo.org/record/4062232#.X3YR9Hn0kuU%E3%80%82
Trajectory test dataset	Nan Miles Xi *et al.*,2019	https://zenodo.org/record/4062232#.X3YR9Hn0kuU%E3%80%82
Lung cancer tumour dataset	Lambrechts *et al.*, 2018	https://gbiomed.kuleuven.be/scRNAseq-NSCLC

Supplementary References

1 McGinnis, C. S., Murrow, L. M. & Gartner, Z. J. DoubletFinder: Doublet Detection in Single-Cell RNA Sequencing Data Using Artificial Nearest Neighbors. *Cell Systems* **8**, 329-337.e324, doi:10.1016/j.cels.2019.03.003 (2019).

2 Wolock, S. L., Lopez, R. & Klein, A. M. Scrublet: Computational Identification of Cell Doublets in Single-Cell Transcriptomic Data. *Cell Syst* **8**, 281-291.e289, doi:10.1016/j.cels.2018.11.005 (2019).

3 Bernstein, N. J. *et al.* Solo: Doublet Identification in Single-Cell RNA-Seq via Semi-Supervised Deep Learning. *Cell Systems* **11**, 95-101.e105, doi:10.1016/j.cels.2020.05.010 (2020).

4 Lun, A. T., McCarthy, D. J. & Marioni, J. C. A step-by-step workflow for low-level analysis of single-cell RNA-seq data with Bioconductor. *F1000Res* **5**, 2122, doi:10.12688/f1000research.9501.2 (2016).

5 Bais, A. S. & Kostka, D. scds: computational annotation of doublets in single-cell RNA sequencing data. *Bioinformatics* **36**, 1150-1158, doi:10.1093/bioinformatics/btz698 (2020).

6 DePasquale, E. A. K. *et al.* DoubletDecon: Deconvoluting Doublets from Single-Cell RNA-Sequencing Data. *Cell Rep* **29**, 1718-1727 e1718, doi:10.1016/j.celrep.2019.09.082 (2019).

7 Kang, H. M. *et al.* Multiplexed droplet single-cell RNA-sequencing using natural genetic variation. *Nat Biotechnol* **36**, 89-94, doi:10.1038/nbt.4042 (2018).

8 Stoeckius, M. *et al.* Cell Hashing with barcoded antibodies enables multiplexing and doublet detection for single cell genomics. *Genome Biol* **19**, 224, doi:10.1186/s13059-018-1603-1 (2018).
