# Supplementary material for: Chord: an ensemble machine learning algorithm to identify doublets in single-cell RNA sequencing data
Source: Commun Biol. 2022 May 30;5:510. doi: 10.1038/s42003-022-03476-9 (PMC9151659; doi:10.1038/s42003-022-03476-9)
Supplement: Supplementary file 3 — Description of Additional Supplementary Files [file 42003_2022_3476_MOESM3_ESM.pdf]

Description of Additional Supplementary Files

File name: Supplementary Data 1
Description: Values of PAUC&PR for all methods in Table 1 in each dataset

File name: Supplementary Data 2
Description: Rank values relative to Figure 2c

File name: Supplementary Data 3
Description: Time-consumption based on cell gradient

File name: Supplementary Data 4
Description: Metadata of Figure 4
